# Risk Factors for Mortality in Children Hospitalized with Severe Malaria in Northern Zambia: A Retrospective Case-Control Study

**DOI:** 10.4269/ajtmh.17-1017

**Published:** 2018-04-23

**Authors:** Matthew M. Ippolito, Luc K. Kamavu, Jean-Bertin Kabuya, Catherine Tente, Edward Chileshe, McBerth Wapachole, Philip E. Thuma, Mbanga Muleba, Mike Chaponda, Modest Mulenga, William J. Moss, for the Southern and Central Africa International Centers of Excellence for Malaria Research

**Affiliations:** 1 Department of Medicine, Johns Hopkins University School of Medicine, Baltimore, Maryland;; 2 Malaria Research Institute, Johns Hopkins Bloomberg School of Public Health, Baltimore, Maryland;; 3 Office of Hospital Administration, Saint Paul’s General Hospital, Nchelenge, Zambia;; 4 Tropical Diseases Research Centre, Ndola, Zambia;; 5 Office of Health Management Information Systems, Saint Paul’s General Hospital, Nchelenge, Zambia;; 6 Ministry of Health, Nchelenge, Zambia;; 7 Macha Research Trust, Macha, Zambia;; 8 Department of Epidemiology, Johns Hopkins Bloomberg School of Public Health, Baltimore, Maryland

## Abstract

Malaria remains a public health crisis in areas where it has resisted control efforts. In Nchelenge District, a high-transmission area in northern Zambia, malaria accounts for more than one-third of pediatric hospitalizations and nearly one-half of hospital deaths in children. To identify risk factors for death due to malaria, we conducted a retrospective, time-matched case-control study of 126 children hospitalized with malaria who died (cases) and 126 children who survived (controls). There were no differences in age, gender, hemoglobin concentration, or prevalence of severe anemia between cases and controls. Children who died were more likely to come from villages located at greater distances from the hospital than children who survived (median 13.5 versus 3.2 km). Each additional kilometer of distance from the hospital increased the odds of death by 4% (odds ratio 1.04, 95% confidence interval 1.01–1.07, *P* < 0.01). Extent of anemia and admission during periods when blood was unavailable for transfusion were associated with early death (*P* ≤ 0.03). Delays in initiation of treatment of severe malaria contribute to the increased odds of death in children referred from more distant health centers, and might be mitigated by transportation improvements, capacity at rural health posts to administer treatment before transfer, hospital triage systems that minimize time to treatment, and reliable blood product stores at referral hospitals.

## INTRODUCTION

Severe malaria, caused by the protozoan *Plasmodium*, is the leading parasitic cause of mortality worldwide. *Plasmodium falciparum*, the most lethal among human malaria parasites, predominates in sub-Saharan Africa where it causes a disconcerting 1,200 child deaths per day concentrated in regions where malaria has proven recalcitrant to control measures or where control measures are tenuous or absent.^1,2^ In Zambia, there were an estimated 3.1 million cases of malaria (severe and uncomplicated) in 2016 among its population of 16.7 million and it was most prevalent in northern Zambia where it persists year round despite the recent scale-up of vector control efforts.^2,3^

Severe malaria can feature one or more of severe anemia (hemoglobin < 5 g/dL), cerebral edema, lactic acidosis, respiratory failure, or shock. In high-transmission, resource-limited settings, malaria evades easy case definition and is often clinically indistinguishable from bacterial meningitis, pneumonia, bacteremia, and other febrile diseases which can conflate or confuse diagnosis.^4^ Only a small proportion (∼2%) of *P. falciparum* infections progress to severe malaria, but in highly malarious regions where children may experience several infections a year, the absolute number of cases is large.^5^ Prompt initiation of treatment is vital. Rapid disease progression is fueled by an exponential expansion of parasites and an exuberant host immune response, and hospital deaths occur most often within 24 hours of admission.^5,6^ The preferred treatment is intravenous artesunate and supportive care as indicated (e.g., fluid resuscitation, blood transfusion, anticonvulsants, and ventilator and vasopressor support), but even with appropriate care, mortality can reach 20%.^5^ Delayed presentation to allopathic health providers on account of logistical constraints or health-seeking behaviors compounds the danger.^7^

Distinguishing factors that contribute to poor outcomes for children with malaria can inform clinical and programmatic strategies to lessen its toll. However, research is limited by the inherent challenges of conducting intrahospital epidemiologic studies in rural centers where malaria prevalence is greatest. We present results of a retrospective, time-matched, case-control study of hospitalized children with malaria who died (cases) and those who survived (controls) at a rural, district-level hospital in a high-transmission region of northern Zambia to identify and evaluate demographic and clinical factors associated with increased risk of death.

## METHODS

### Study site.

The study was conducted in Nchelenge District, a high malaria transmission area of northern Zambia. Since 2012, the National Malaria Elimination Program under the Ministry of Health have overseen bed net distributions and indoor residual spray campaigns in the study area, although little decrease in malaria burden has followed.^3^ Community prevalence by rapid diagnostic tests is estimated to be ∼50%.^8^ In 2016, there were 1,128 adult and child hospital admissions for malaria, accounting for 35% of all pediatric hospital admissions and 38% of all pediatric deaths in the hospital. The malaria case fatality rate was 80 and 65 deaths per thousand adult and pediatric hospitalizations for malaria in 2016 and 2017, respectively.

A single 175-bed hospital staffed by three physicians and one midlevel provider serves the district and surrounding areas. The hospital is a referral center for 11 rural health centers that lie 1.5 to > 40 km away, including an island community 30 km away. The rural health centers are neither outfitted with the means to initiate specific therapy for severe malaria, which includes intravenous artesunate, nor are alternatives such as rectal artesunate and intramuscular artemether available.^5^ Children judged by health center providers to require care for severe malaria are transferred to the hospital. Transportation from health centers to the hospital is challenging: patients’ families must arrange transport for a fee, and road conditions are poor.

Some patients do not survive transfer from the rural health center to the hospital or from the hospital outpatient department to the ward, and are recorded in ward registers as dead on arrival. Administration of intravenous artesunate, blood transfusion, and other supportive care can only occur once a child has been transferred to the ward.

### Study design.

This was a retrospective, time-matched, case-control study of children with severe malaria admitted to the hospital who died (cases) or survived (controls). Cases were matched 1:1 with controls admitted on or near the same date to account for differences in health-care providers, stockouts of blood products or other hospital resources, and other unmeasured temporal confounders such as road conditions, which vary between the rainy and dry seasons and were hypothesized to impact time to treatment. If more than one control was identified for a given day, the patient admitted closest in time to the case was selected. The study was approved by the Johns Hopkins Bloomberg School of Public Health Institutional Review Board as part of the International Centers of Excellence for Malaria Research study and locally by the Tropical Diseases Research Centre Ethics Review Committee located in Ndola, Zambia.

### Data collection.

Case-control data were collected from hospital registers and laboratory logbooks for children admitted to the hospital between January 2016 and October 2017. Severe malaria was determined by the discharge diagnosis in the register, and the primary outcome of the study (survival or death) was determined by the recorded disposition in the register. All patients admitted to the children’s ward with severe malaria who died were included. Those who did not survive were further stratified by those who died before treatment could be administered (dead on arrival) and those who died after they were admitted and underwent treatment. Patient charts of children who died were frequently unavailable, precluding chart extraction of data.

Hospital epidemiologic data were collected from the hospital Health Management Information Systems database (current to April 2017) and hospital registers. Distances from villages to the hospital were provided by the district office of the Zambian Ministry of Health.

All hemoglobin values were presumed to be pretransfusion values. Hospital practice is to require documented hemoglobin level before transfusion, and patients infrequently undergo more than a single laboratory evaluation. We use the term initial hemoglobin because not all children were subsequently transfused (typical transfusion threshold is ≤ 5.0 g/dL).

### Statistical analysis.

Matched case-control data were analyzed by conditional logistic regression. Linear and logistic regression models were applied for unmatched adjusted analyses of early death. We hypothesized that the relationship between village-to-hospital distance and survival would be partially mediated by the degree of anemia as patients with delayed presentation are more likely to have low mean hemoglobin concentrations due to disease progression. Mediation analysis was carried out to estimate the percent contribution of hemoglobin concentration to the total indirect effect of distance from the hospital on survival. Sensitivity analyses were performed, one excluding patients from the two most remote villages and their matched controls to account for outlying distances and another introducing a covariate designating admission during a blood stockout period to account for the five study pairs mismatched on that variable. A subgroup analysis of patients who were dead on arrival to the children’s ward was performed to examine risk factors of early death. In a cross-sectional design, these patients were compared with cases who survived the initial admission period but subsequently died. The frequencies of severe malaria hospitalization and death outcomes in relation to blood stockout periods were analyzed using Poisson regression. Statistical significance was prespecified to a threshold *P* value of 0.05 and two-sided α. Analyses were carried out using Stata 14 (StataCorp, College Station, TX).

## RESULTS

### Study participants.

We identified 126 children admitted to the children’s ward with a diagnosis of severe malaria who died during the period January 2016 through October 2017. Most (89%) matched control patients were admitted within 1 day of the case patients, and of those, more than half (54%) were admitted the same day. Eleven were admitted within 2 days of the matched control and three within 3 days. Five of the 126 case-control pairs were mismatched on whether there was a blood stockout on the day of admission. One case was missing age and gender, but hemoglobin and village data were available. Data on village distance to the hospital were available for all except nine control and 18 case patients. Hemoglobin concentrations were unavailable for 49 (39%) control and 41 (33%) case children. Missingness was mainly due to hemoglobin measurements done at rural health centers prior to hospital transfer, hence results were not recorded in laboratory logbooks. Children whose hemoglobin measurements were not available were similar in age, gender, and distance from the hospital to those for whom hemoglobin data were available.

### Age, gender, hemoglobin, and severe anemia.

There were no statistically significant differences in age, gender, initial hemoglobin concentration, or prevalence of severe anemia between children who survived and those who died (Table 1). The median age of study children was 1 year 11 months, ranging from 2 months to 18 years. Slightly more than half (53%) were girls. The mean initial hemoglobin concentration of all children was 5.4 g/dL (standard deviation 2.8 g/dL). Prevalence of severe anemia was 61% among cases and 47% among controls, but the difference was not significant after adjustment for age and gender (odds ratio [OR] 2.04, 95% confidence interval [CI] 0.87–4.79, *P* = 0.10).

**Table 1:** Characteristics of children admitted to the hospital with a diagnosis of severe malaria who died (cases) and survived (controls)

Characteristic	Cases	Controls	*P* Value
	*n* = 126	*n* = 126	
Female, *n* (%)	66 (52.8)	67 (53.2)	0.70
Age, months, median (IQR)	21 (12–36)	24 (13–36)	0.85
Hemoglobin concentration (g/dL), mean (SD)*	5.0 (2.8)	5.8 (2.7)	0.16
Presence of severe anemia, *n* (%)	52 (61.2)	36 (46.8)	0.10
Hemoglobin < 3 g/dL, *n* (%)	20 (23.5)	10 (13.0)	0.20
Distance from home village to hospital (km), median (IQR)	13.5 (2.3–26.0)	3.2 (1.5–14.0)	< 0.01
Presentation during blood product stockout, *n* (%)	27 (21.4)	22 (17.5)	0.99

IQR = interquartile range; SD = standard deviation.

The *P* values were estimated by conditional logistic regression of matched case–control data.

* Initial values measured on presentation.

### Distance from hospital.

**Figure 1.**
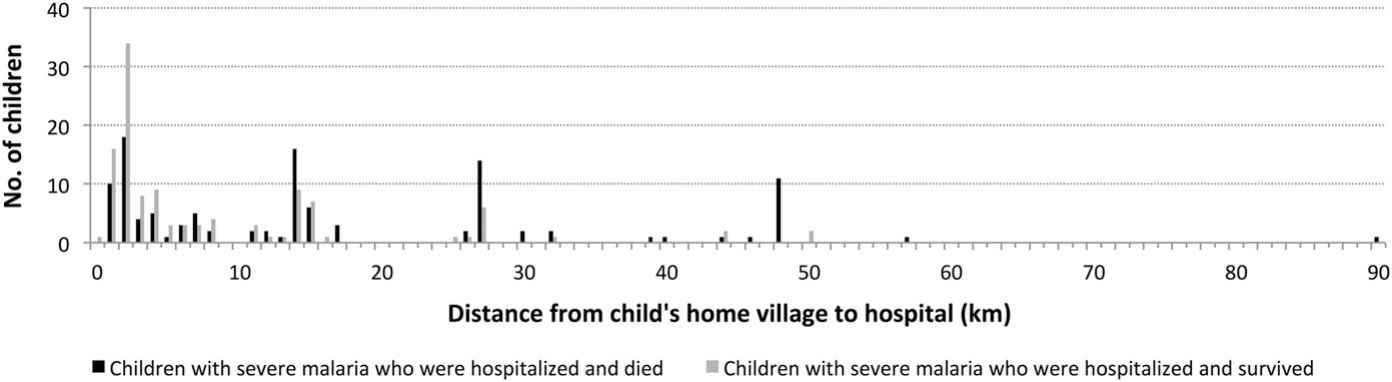
Distribution of home village-to-hospital distances among children with severe malaria who were hospitalized and died (cases) or survived (controls).

Children came from 73 villages situated between 1.5 and 89.6 km from the hospital. One child (control) lived within the hospital compound and one child (case) was from the island community located 30 km away. Those with severe malaria who did not survive were more likely to come from distant villages than those who survived (Figure 1). Adjusted for age and gender, every 1 km of distance conferred a 4% increase in odds of death (OR 1.04, 95% CI 1.01–1.07, *P* < 0.01). Mediation analyses estimated that hemoglobin concentration accounted for only 4% of the contribution of distance from the hospital to death among all case patients, and 28% among those recorded as dead on arrival to the ward. In a sensitivity analysis excluding cases from the two most outlying villages and their matched controls, the association between distance and death remained statistically significant (OR 1.04, 95% CI 1.01–1.07, *P* < 0.01). The same was true when introducing a covariate for admission during a stockout period to account for the five mismatched pairs.

### Dead on arrival to ward.

**Figure 2.**
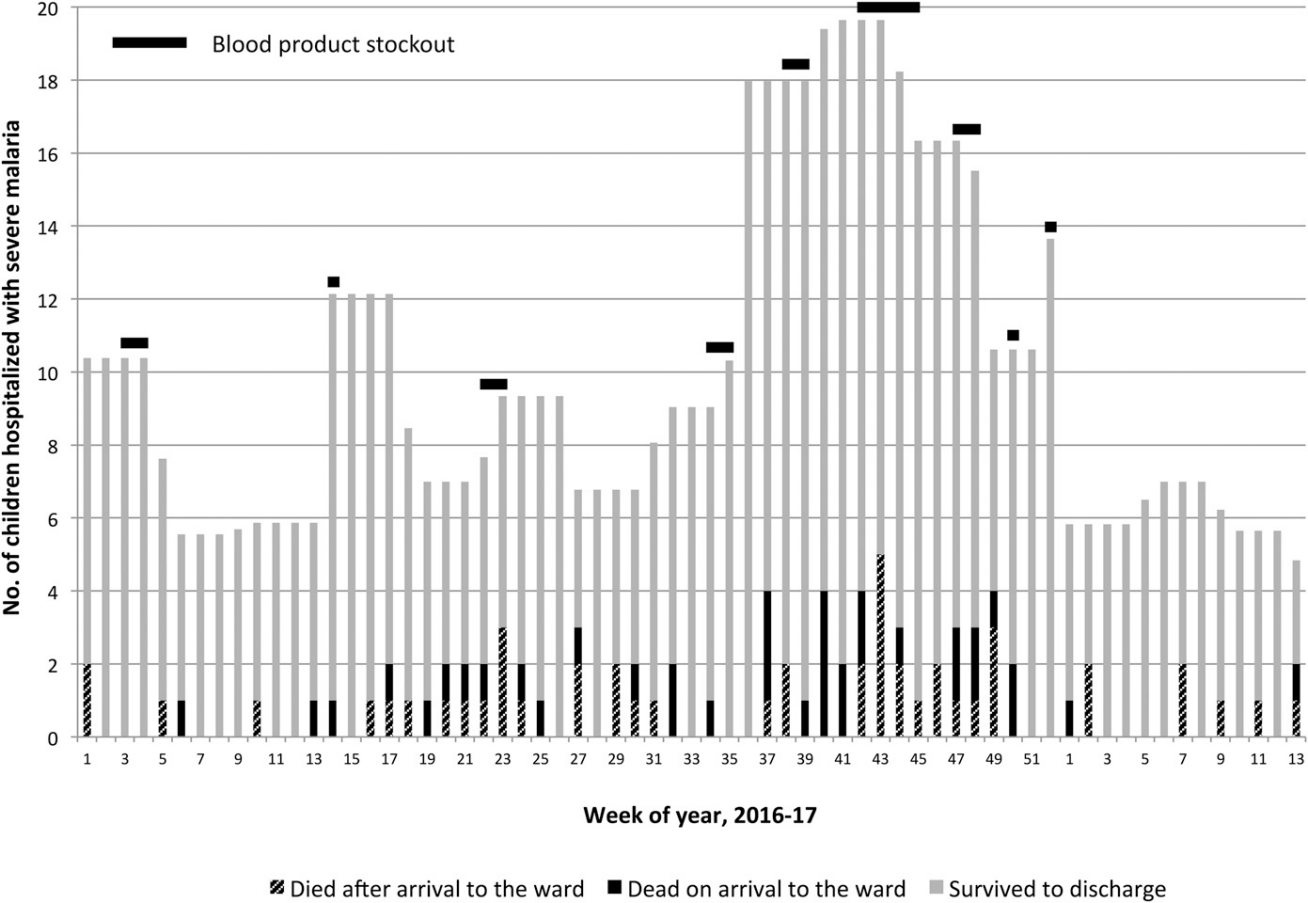
Outcomes of hospitalization among children with severe malaria admitted to the pediatric ward showing periods of blood product stockouts.

Among the 126 children with severe malaria who died, 47 (37%) were dead on arrival to the ward. Patients dead on arrival did not differ in age or gender from those who died after arrival to the ward (Table 2). However, they came from more distant villages (median 14 versus 11 km, *P* = 0.05), anemia was more severe (mean hemoglobin 3.8 versus 5.6 g/dL, *P* = 0.01), and a higher proportion were admitted when blood was unavailable for transfusion (31% versus 17%, *P* = 0.03) (Table 2, Figure 2).

**Table 2:** Characteristics of children with severe malaria who were dead on arrival to the ward and those who died after arrival to the ward

Characteristic	Dead on arrival	Died after arrival	*P* Value
	*n* = 47	*n* = 79	
Female, *n* (%)	26 (55.3)	40 (51.3)	0.42
Age, months, median (IQR)	21 (12–36)	22 (12–36)	0.09
Hemoglobin concentration (g/dL), mean (SD)*	3.8 (1.6)	5.6 (3.1)	0.01
Presence of severe anemia, *n* (%)	19 (73.1)	33 (55.9)	0.06
Hemoglobin < 3 g/dL, *n* (%)	7 (26.9)	13 (22.0)	0.20
Distance from home village to hospital (km), median (IQR)	14.0 (2.3–26.6)	11.0 (3.2–15.5)	0.05
Presentation during blood product stockout, *n* (%)	14 (31.1)	13 (16.5)	0.03

IQR = interquartile range; SD = standard deviation.

*P* values were estimated by logistic regression of cross-sectional (cases only) data.

* Initial values measured on presentation.

### Blood inventory stockouts.

Within the study period, there were 12 whole blood stockouts ranging from 3 days to 3 weeks, totaling 68 days. Stockouts were more common during months with greater-than-average daily pediatric admissions for malaria (OR 7.1, 95% CI 4.5–11.3, *P* < 0.01). During stockout periods, the incidence of death among children with malaria was once in 3 days, compared with one death every 6 days during periods when blood was in supply (daily mean number of deaths 0.30 versus 0.18, *P* = 0.02), but the number of deaths as a proportion of admissions did not differ (0.13 versus 0.13, *P* = 0.88).

## DISCUSSION

This case-control study of children hospitalized with malaria in a high-transmission setting of northern Zambia found that the strongest predictor of mortality was distance from the patient’s village to the hospital. Each kilometer of distance from the hospital was accompanied by a 4% increase in the odds of death. Low hemoglobin and hospitalization during blood inventory stockouts were associated with early death, defined as those recorded as dead on arrival to the children’s ward.

Distance to health centers is a well-documented risk factor for child mortality in sub-Saharan Africa, although few studies have examined its influence on malaria fatality.^9–12^ Long distance delays time to the first dose of intravenous artesunate, time to blood transfusion, and time to other supportive care. Living a greater distance from the hospital may be associated with other factors that affect malaria health outcomes, including delayed health-care–seeking behaviors.^7,13^

The young ages of the patients, and the prevalence and profundity of anemia, were commensurate with a high malaria transmission environment.^14,15^ In contrast to prior studies of severe malaria conducted elsewhere in sub-Saharan Africa, we did not find an association between age and mortality, perhaps explained by the overall low median age of our study population (< 2 years) relative to previously studied populations.^16–18^ Most children (85%) were younger than 5 years and age-related factors (e.g., premunition) may have been attenuated because of the narrow age range of the study children.

Blood stockouts were more common during peaks in hospital admissions for malaria. The number of deaths among hospitalized children with malaria was higher during stockout periods, but modeling showed this to be explained wholly by the increased number of admissions and not the depletion of blood stores.

There were two unexpected findings related to the role of anemia. First, we detected no significant difference in hemoglobin concentrations between those who survived and those who died. Sample size limitations imposed by missingness in hemoglobin data and potential instances of misclassification of the cause of death leading to artificial inflation of the mean hemoglobin relative to controls are possible explanations for this finding. Based on data from a pivotal trial of artesunate versus quinine for severe malaria,^19^ hemoglobin appears to influence survival only at concentrations < 3 g/dL.^20^ Twice as many cases as controls in our sample had such low hemoglobin concentrations, though the difference did not reach the preset significance threshold (*P* = 0.20).

The second unexpected finding was that hemoglobin concentration explained only a small portion (4%) of the effect of distance on malaria death. We hypothesized that greater distance from the hospital would correspond with delay in presentation and thereby more profound anemia, and ultimately a higher risk of death, as the illness progressed unchecked. Although we did not observe this in the overall sample, the association was observed among children brought in dead to the ward. In these children, hemoglobin concentration accounted for > 25% of the estimated effect of distance on death, consistent with the additional finding of an association between children who were dead on arrival to the ward and admission during a blood stockout period. Hemoglobin testing and subsequent blood transfusion, if indicated and possible, are often done between the time the child arrives to the hospital and is registered on the ward. Our findings suggest that those who did not survive the interval between presentation to the hospital and registration on the ward often died for lack of transfusion. These represent children who never had the opportunity to receive treatment and supportive care. Had they survived long enough to undergo treatment, it is likely that some would have recovered and survived to discharge, implying that ensuring reliable blood inventories would improve malaria survival.

There are limitations to this study. Reliance on hospital registers precluded a rigorous case definition of severe malaria and death due to malaria, introducing potential misclassification bias. However, given the high background prevalence of malaria in the catchment area, ready availability of malaria diagnostics, and experienced hospital staff, we believe this is unlikely to have significantly influenced the study’s results. Furthermore, there is precedent for our case definition; for example, the Severe Malaria in African Children network enrolled any child with malaria sick enough to be hospitalized.^21^ Similarly, we were unable to ascertain clinical syndromes (e.g., cerebral malaria, lactic acidosis) nor could we account for children with coinfections or incidental parasitemia unrelated to their illness. A hospital-based study such as this is unable to account for children who die at home from complications of malaria, die at rural health centers or healers, or die en route to the hospital, who constitute most severe malaria deaths in some settings.^22,23^ There are potential unmeasured confounders and mediators of the effect of distance on malaria death, including factors related to delays in care such as health-seeking behavior and knowledge, attitudes and practices around malaria, time to first dose of artesunate, and time to blood transfusion.

A precept of malaria control is prompt diagnosis and treatment to reduce deaths from severe malaria and to diminish the infectious reservoir. National and local malaria control programs prioritize surveillance of and response to severe malaria morbidity and mortality, but these priorities are often detrimentally overlooked by the research community where clinical trials of interventions for severe malaria are sparse and progress thereby slow.^24^ In the meantime, investments in transportation infrastructure (e.g., road improvements) to reduce transfer times to the hospital, hospital triage systems that expedite treatment on arrival for patients with severe malaria, and maintenance of responsive supply chains for blood inventories to avert stockouts during periods of high malaria incidence would temper case fatality. Stationing ambulances at remote health posts and furnishing them with the capacity to initiate pre-transfer treatment of severe malaria, such as intramuscular or rectal artesunate, would be lifesaving.

## References

[1] Black RE 2010 Global, regional, and national causes of child mortality in 2008: a systematic analysis. Lancet 375: 1969–1987. 20466419 10.1016/S0140-6736(10)60549-1

[2] World Health Organization, 2017 *World Malaria Report*. Geneva, Switzerland: WHO Global Malaria Programme.

[3] Mukonka VM 2014 High burden of malaria following scale-up of control interventions in Nchelenge District, Luapula province, Zambia. Malar J 13: 153. 24755108 10.1186/1475-2875-13-153 PMC4016669

[4] Sypniewska P Duda JF Locatelli I Althaus CR Althaus F Genton B, 2017 Clinical and laboratory predictors of death in African children with features of severe malaria: a systematic review and meta-analysis. BMC Med 15: 147. 28768513 10.1186/s12916-017-0906-5 PMC5541406

[5] World Health Organization, 2014 Severe malaria. Trop Med Int Health 19 (Suppl 1): 7–131. 25214480 10.1111/tmi.12313_2

[6] Ioannidis LJ Nie CQ Hansen DS, 2014 The role of chemokines in severe malaria: more than meets the eye. Parasitology 141: 602–613. 24476686 10.1017/S0031182013001984 PMC3962270

[7] Sundararajan R Mwanga-Amumpaire J Adrama H Tumuhairwe J Mbabazi S Mworozi K Carroll R Bangsberg D Boum Y 2nd Ware NC, 2015 Sociocultural and structural factors contributing to delays in treatment for children with severe malaria: a qualitative study in southwestern Uganda. Am J Trop Med Hyg 92: 933–940. 25802438 10.4269/ajtmh.14-0784 PMC4426580

[8] Pinchoff J Chaponda M Shields TM Sichivula J Muleba M Mulenga M Kobayashi T Curriero FC Moss WJ, Southern Africa International Centers of Excellence for Malaria Research, 2016 Individual and household level risk factors associated with malaria in Nchelenge district, a region with perennial transmission: a serial cross-sectional study from 2012 to 2015. PLoS One 11: e0156717. 27281028 10.1371/journal.pone.0156717 PMC4900528

[9] Schoeps A Gabrysch S Niamba L Sie A Becher H, 2011 The effect of distance to health-care facilities on childhood mortality in rural Burkina Faso. Am J Epidemiol 173: 492–498. 21262911 10.1093/aje/kwq386

[10] Kadobera D Sartorius B Masanja H Mathew A Waiswa P, 2012 The effect of distance to formal health facility on childhood mortality in rural Tanzania, 2005–2007. Glob Health Action 5: 19099. 10.3402/gha.v5i0.19099 PMC3495250 23151364

[11] Abdul-Aziz AR Harris E Munyakazi L, 2012 Risk factors in malaria mortality among children in northern Ghana: a case study at the Tamale teaching hospital. Int J Business Social Res 2: 35–45.

[12] Kazembe LN Kleinschmidt I Sharp BL, 2006 Patterns of malaria-related hospital admissions and mortality among Malawian children: an example of spatial modelling of hospital register data. Malar J 5: 93. 17067375 10.1186/1475-2875-5-93 PMC1635723

[13] Müller O Garenne M Kouyate B Becher H, 2003 The association between protein-energy malnutrition, malaria morbidity and all-cause mortality in West African children. Trop Med Int Health 8: 507–511. 12791055 10.1046/j.1365-3156.2003.01043.x

[14] Modiano D Sirima BS Sawadogo A Sanou I Pare J Konate A Pagnoni F, 1998 Severe malaria in Burkina Faso: influence of age and transmission level on clinical presentation. Am J Trop Med Hyg 59: 539–542. 9790426 10.4269/ajtmh.1998.59.539

[15] Snow RW Guerra CA Noor AM Myint HY Hay SI, 2005 The global distribution of clinical episodes of *Plasmodium falciparum* malaria. Nature 434: 214–217. 15759000 10.1038/nature03342 PMC3128492

[16] Varandas L Julien M Van Lerberghe W Goncalves L Ferrinho P, 2000 Independent indicators of outcome in severe paediatric malaria: maternal education, acidotic breathing and convulsions on admission. Ann Trop Paediatr 20: 265–271. 11219163 10.1080/1465328002003852

[17] Bronzan RN 2007 Bacteremia in Malawian children with severe malaria: prevalence, etiology, HIV coinfection, and outcome. J Infect Dis 195: 895–904. 17299721 10.1086/511437

[18] Mabeza GF Biemba G Brennan AG Moyo VM Thuma PE Gordeuk VR, 1998 The association of pallor with haemoglobin concentration and mortality in severe malaria. Ann Trop Med Parasitol 92: 663–669. 9924545 10.1080/00034989859122

[19] Dondorp A Nosten F Stepniewska K Day N White N, South East Asian Quinine Artesunate Malaria Trial group, 2005 Artesunate versus quinine for treatment of severe falciparum malaria: a randomised trial. Lancet 366: 717–725. 16125588 10.1016/S0140-6736(05)67176-0

[20] von Seidlein L 2012 Predicting the clinical outcome of severe falciparum malaria in African children: findings from a large randomized trial. Clin Infect Dis 54: 1080–1090. 22412067 10.1093/cid/cis034 PMC3309889

[21] Taylor T 2006 Standardized data collection for multi-center clinical studies of severe malaria in African children: establishing the SMAC network. Trans R Soc Trop Med Hyg 100: 615–622. 16551469 10.1016/j.trstmh.2005.09.021 PMC1459261

[22] Mudenda SS Kamocha S Mswia R Conkling M Sikanyiti P Potter D Mayaka WC Marx MA, 2011 Feasibility of using a World Health Organization-standard methodology for sample vital registration with verbal autopsy (SAVVY) to report leading causes of death in Zambia: results of a pilot in four provinces, 2010. Popul Health Metr 9: 40. 21819583 10.1186/1478-7954-9-40 PMC3160933

[23] Greenwood BM Bradley AK Greenwood AM Byass P Jammeh K Marsh K Tulloch S Oldfield FS Hayes R, 1987 Mortality and morbidity from malaria among children in a rural area of the Gambia, West Africa. Trans R Soc Trop Med Hyg 81: 478–486. 3318021 10.1016/0035-9203(87)90170-2

[24] Maitland K, 2016 Severe malaria in African children–the need for continuing investment. N Engl J Med 375: 2416–2417. 28002698 10.1056/NEJMp1613528 PMC6858851

